# Improvement of Insulin Sensitivity Increases Pregnancy Rate in Infertile PCOS Women: A Systemic Review

**DOI:** 10.3389/fendo.2021.657889

**Published:** 2021-03-30

**Authors:** Yuqi Liu, Juan Li, Zhe Yan, Dan Liu, Jinfang Ma, Nanwei Tong

**Affiliations:** ^1^ Department of Endocrinology and Metabolism, West China Hospital of Sichuan University, Chengdu, China; ^2^ Laboratory of Diabetes and Islet Transplantation Research, Center for Diabetes and Metabolism Research, West China Hospital of Sichuan University, Chengdu, China; ^3^ Department of Obstetrics and Gynecology, Nantong Rich Hospital of Nantong University, Nantong, China

**Keywords:** polycystic ovary syndrome (PCOS), insulin sensitivity, review – systematic, randomized controlled trials (RCT), insulin sensitizers

## Abstract

**Background:**

Polycystic ovary syndrome (PCOS) is the most common cause of infertility in reproductive-age women. Insulin increases steroidogenesis, deranges granulosa cell differentiation, and affects follicle growth. However, results from randomized control trials (RCTs) were heterogeneous, and little strong evidence associated actual achievement of insulin sensitivity (IS) improvement with reproductive outcomes.

**Objectives:**

To identify evidence of the reproductive benefit of IS improvement in infertile PCOS women by analyzing eligible RCTs.

**Search Strategy:**

Different search strategies with unlimited keywords, including treatment, therapy, intervention, polycystic ovary syndrome/PCOS, insulin resistance, pregnancy, conceive, live birth, and randomized controlled trials/RCT were used in databases including Pubmed, Embase, and Web of Science to November 20th, 2021.

**Data Collection and Analysis:**

Two authors independently abstracted study details and assessed study quality.

**Main Results:**

Ten RCTs that covered different races and met the inclusion criteria were included for analysis and discussion. Clinical pregnancy rate was increased in infertile PCOS women when they had significant improvement of IS after treatment regardless of the various interventions (non-surgical). The benefits of IS improvement appeared superior in PCOS women without severe obesity. The effect of IS improvement on pregnancy rate was independent of the change of BMI.

**Conclusions:**

Nonsurgical therapeutic strategies that promote superior IS improvement may aid infertile PCOS women to increase their possibility of successful pregnancy regardless of the various interventions. The improvement of IS might be more important than the reduction of BMI in the improvement of pregnancy rate in infertile PCOS women.

## Introduction

Polycystic ovary syndrome (PCOS) is the most common endocrine disease in reproductive-age women, characterized by hyperandrogenism and olig-anovulation. It is considered a metabolic disorder because it is associated with high rates of insulin resistance (IR), dyslipidemia, obesity, and other metabolic abnormalities. The prevalence of PCOS can reach 18% in reproductive-age women (1) and those with PCOS have a higher risk of infertility and other health issues (2).

Depending on the pregnancy needs of individuals, therapies for women with PCOS differ. For women who do not desire conception, the aim of treatment is to improve symptoms including acne, hirsutism, and menstrual disorder. Regarding adult infertile women with PCOS, achieving successful pregnancy and live birth are the primary objectives. In PCOS patients with oligo-anovulation, the first-line ovulation induction agents are clomiphene citrate (CC), letrozole (LZ), and metformin (3). CC is an antiestrogen, nonsteroidal compound that can induce ovulation in anovulatory women (4). LZ is an aromatase inhibitor that inhibits the conversion of adrenal androgens to estrogens. It is generally employed for breast cancer therapy (5), and has been found to function as an ovulation induction agent. Metformin, a first-line drug recommended by guidelines for the treatment of diabetes (6), was shown to induce ovulation and improve pregnancy rate in infertile women with PCOS (7, 8), although not as effectively as CC, according to a meta-analysis (9).

The mechanism underlying metformin’s effects in PCOS women remains unclear. Metformin improves insulin sensitivity (IS) in the liver and peripheral tissues, which may represent the mechanism explaining its effect in improving pregnancy in PCOS women. The prevalence of IR was reported as 44%–70% in women with PCOS when assessed by surrogate markers (10). PCOS has a multifactorial etiology including intra-uterine, genetic and environmental factors. Familial aggregation studies indicated that PCOS is an inherited disorder and gene variants associated with IR have been demonstrated in PCOS patients. And intra-uterine growth restriction (IUGR) and small for gestational age (SGA) might cause excess glucocorticoids which increased the risk of obesity and hyperinsulinemia during childhood and finally contributed to developing insulin resistance in PCOS women (11). Supraphysiological doses of insulin were found to increase steroidogenesis, derange granulosa cell differentiation, and affect follicle growth (12). However, evidence regarding reproductive outcomes of insulin-sensitizing drugs in PCOS was inconsistent (13) and few analyses focused on actual changes in IS after treatment, which reflects whether these drugs achieve improvement of IR in PCOS women. In another word, the inconsistencies may have been because of failure to achieve improvement of IS. Additionally, some randomized control trials (RCTs) observed beneficial effects of CC and other nondiabetic drugs on IS and also on clinical pregnancy rate (14, 15). Taken together, these observations suggest achieving improvement of IS may be the actual factor that promotes an increased rate of pregnancy.

In this review, we summarized data from RCTs that reported post-treatment changes in IS, with the objective of analyzing the correlation between improvement of IS and pregnancy rate in infertile women with PCOS who underwent various nonsurgical interventions, and exploring the reproductive outcomes of insulin-sensitizers, such as pioglitazone and exenatide, in PCOS women.

## Methods

### Systematic Review Methodology

The systematic review was conducted according to the guidelines from PRISMA. We searched electronic databases, including MEDLINE from PubMed, Embase, and Web of Science with different search strategies and no limiting keywords, including treatment, therapy, intervention, polycystic ovary syndrome/PCOS, insulin resistance, pregnancy, conceive, live birth, and randomized controlled trials/RCT, to November 20, 2020.

Articles were screened by title and abstract. We established the following inclusion/exclusion criteria according to PICOS. 1) Population (P): reproductive-age women who were infertile because of PCOS rather than other reproductive diseases, and who were not complicated with diabetes or other endocrine disorders. 2) Interventions (I): administration of various reasonable nonsurgical interventions including lifestyle modification, monotherapies, and combined therapies was acceptable. Studies that used assisted reproductive technology or any surgical interventions were excluded. However, at least one parameter pertaining to IS (fasting blood glucose/insulin ratio, insulin sensitivity index, homeostasis model assessment of insulin resistance (HOMA-IR)) was reported before and after treatment. 3) Comparison/control (C): All head-to-head and placebo-controlled RCT were included. 4) Outcomes (O): clinical pregnancy rate and/or live birth rate. 5) Study (S): randomized controlled trials. 6) Additional inclusion/exclusion criteria: To assess IR, baseline fasting blood glucose in the different treatment groups of the included studies was not significantly different. And only English-language literature was included due to the language barrier.

To include as many relevant clinical trials as possible and without bias, the criteria did not limit treatment duration or sample size. The effects of treatment duration and sample size will be discussed.

Two authors independently abstracted study details and assessed the quality of RCTs in a blinded fashion.

### Definition of Insulin Sensitivity and its Improvement After Treatment

Studies evaluated IS or IR according to different surrogate parameters, including fasting blood glucose/fasting serum insulin ratio, insulin sensitivity index, and HOMA-IR. Although some RCTs reported insulin levels (14–21) before and after treatment, and blood glucose levels, they did not report parameters that reflect IS or IR. Therefore, these trials were excluded from this review.

Each included trial reported whether there was a significant change in the IS parameter before and after treatment, and whether it was significantly different between the groups in the trial. The group with significantly increased insulin sensitivity index or reduced HOMA-IR compared with other groups in the same trial was considered to have achieved an improvement of IS. Because of the lack of uniformity in the units of measurement of IS criteria, comparisons were only conducted within a single trial, and could not be conducted with meta-analysis.

### Criteria for Overweight and Obesity Among Different Races

Owing to differences in race, diagnostic criteria for overweight and obesity differ. In America and most Western countries, BMI between 25.0 and 29.9 is considered overweight, BMI>30.0 is obese, and BMI≥35.0 is considered severely obese (22). However, in Asian populations, BMI between 23.0–27.5 is overweight, BMI>27.5 is obese, and BMI≥32.5 is severely obese (23). In this review, we followed the above criteria and classified PCOS women from the included trials as overweight, obese, or severely obese according to race.

### Assessment for Quality of RCTs

The included RCTs were evaluated for quality by using the Cochrane Risk of Bias tool with RevMan 5.4. The assessment consists of seven aspects: random sequence generation, allocation concealment, blinding of participants and personnel, blinding of outcome assessment, incomplete outcome data, selective outcome reporting, and other bias.

## Results

**Figure 1:**
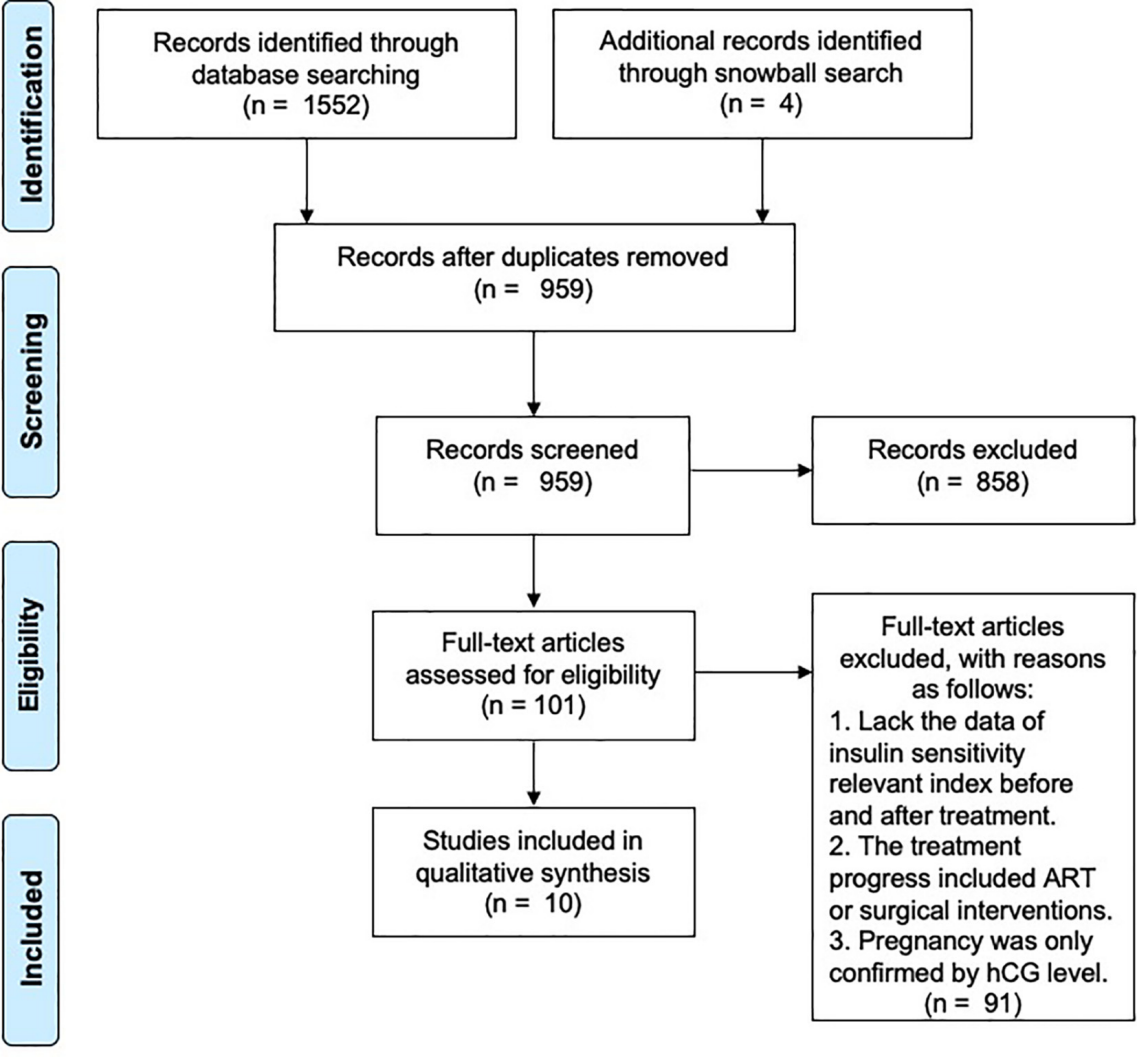
Flow chart showing literature search and study selection.

The preliminary search identified 959 unique citations. After screening on title and abstract, 101 eligible studies were identified by full text. In total, 10 studies were included in the final selection (**Figure 1**).

Ten RCTs from seven countries that covered different races and met the inclusion criteria were included for analysis (24–33). Sample sizes of included RCTs ranged from 25 to 626 (**Table 1**). To avoid bias, we included RCTs with small sample sizes, the impact of which is discussed below.

**Table 1:** The sample size, treatment duration, the formula and units of the IS parameters, and the basic condition of subjects in the included RCTs.

Trial No.	Study	Intervention Group (Subjects number in each group)	Sample size	Treatment Duration	CC resistant or not	BMI before treatment	Classification according to BMI^1^	Parameter of IS	The formula and Unit of the Parameter that reflects IS
1	Wang et al. (31)	Metf+CC (N=33)	78	up to 6 months	unknown	25.74±6.37	Over weight	HOMA-IR	FPG (mmol/L) × FINS (mIU/L) /22.5
		Exenatide+CC (N=45)				26.26±5.71			
2	Agrawal et al. (24)	Metformin+CC (N=60)	120	6 months	unknown	27.38±3.92	Over weight	HOMA-IR	mmol/L
		Metformin+MYO+CC (N=60)				27.71±3.60			
3	Ortega-González et al. (30)	Metformin (N=18)	47	6 months	unknown	34.1±1.6	Obese	HOMA-IR	FPG (mmol/L) × FINS (μIU/ml) /22.5
		Pioglitazone (N=17)				32.2±1.0			
4	Liu et al. (29)	24w Metf (N=80)	158	24 weeks	unknown	28.29±1.86	Obese	HOMA-IR	FPG (mmol/L) × FINS (mIU/L) /22.5
		12w Exenatide switch to 12w Metf (N=78)				29.16±3.11			
5	El Sharkwy and Sharaf El-Din (33)	Metf+CC (N=136)	274	3 months	CC-resistant	34.4±3.4	Severe Obese	HOMA-IR	FPG (mmol/L) × FINS (mIU/L) /22.5
		Metf+CC+L-carnitine (N=138)				35.5±3.2			
6	Legro et al. (27)	Metformin (N=208)	626	up to 6 months	unknown	35.6±8.5	Severe obese	Change in HOMA-IR	FPG (mg/dl) × FINS (μU/ml) /405
		CC (N=209)				36.0±8.9			
		Metformin+CC (N=209)				34.2±8.4			
7	Karimzadeh et al. (26)	Metformin (N=100)	200	3 months	unknown	28.80±3.18	Over weight	FPG/Insulin	The units were not reported
		Placebo (N=100)				29.49±4.75			
8	Yarali et al. (32)	Metformin (N=10)	25	6 weeks	CC-resistant	28.6±4.0	Over weight	FPG/Insulin	FPG (mmol/L) /FINS (pmol/L)
		Placebo (N=15)				29.6±4.8			
9	George et al. (25)	Metf+CC (N=30)	60	6 months	CC-resistant	25.7±3.9	Over weight	FPG/insulin	FPG (mg/dl) /FINS (μIU/ml)
		hMG (N=30)				25.9±2.9			
10	Legro et al. (28)	Lifestyle modification (N=50)	149	16 weeks	unknown	35.1±4.6	Severe obese	insulin sensitivity index	10,000/square root of (FPG x FINS) x (mean glucose x mean insulin during OGTT)
		OCPs (N=49)				35.1±4.2			
		Combination (N=50)				35.5±4.4			

1, The Asian criteria of overweight is BMI between 23-27.5, obesity is BMI>27.5, and severe obesity is BMI>=32.5.

BMI, Body mass index; CC, clomiphene citreate; Metf, Metformin; MYO, myoinositol; hMG, human menopausal gonadotropin; OCPs, Oral contraceptive pills; HOMA-IR, Homeostatic Model Assessment of insulin resistance; IS, Insulin sensitivity; FPG, Fasting Plasma Glucose; FINS, Fasting Insulin; OGTT, Oral Glucose Tolerance Test; RCT, Randomized Controlled Trial.

Five RCTs used the HOMA-IR index as the parameter for evaluating IS (Trial No.1-6 in Tables) (24, 27, 29–31, 33), whereby larger value indicates more IR (less IS), whereas three studies reported the fasting plasma glucose (FPG)/insulin (INS) ratio (Trial No.7-9) (25, 26, 32), whereby larger value indicates improved IS. Only Legro et al. (Trial No.10 in Tables) used the insulin sensitivity index (28) (**Table 1**). Although the various parameters and units of measurement employed by each study made it difficult to compare IS status among different trials, changes in IS could be compared within a given trial (**Table 1**).

Only four trials among the included RCTs reported live birth rate (Trial No.2, 3, 6, 10) (24, 27, 28, 30), although all ten reported clinical pregnancy rate (**Table 2**). Therefore, the primary outcome of this review was pregnancy rate, and live birth rate was analyzed when it was reported.

**Table 2:** The pre- and post- BMI, insulin sensitivity parameters in the RCTs and pregnancy rate and live birth rate in the included RCTs.

Trial No.	Study	Intervention Group	BMI before treatment	BMI after treatment	Sigificant change in BMI among groups	Parameter of IS	Parameter of IS before treatment	Parameter of IS after treatment^1^	Significant change of IS among groups^2^	Pregnancy rate	Significant changes of Pregnancy rate among groups^2^	Live birth rate	Significant change in Live birth rate among groups^2^
1	Wang et al. (31)	Metf+CC (N=33)	25.74±6.37	-		HOMA-IR	6.93±0.69	5.79±0.58	Y	9/33 (27.3%)	Y	-	-
		Exenatide+CC (N=45)	26.26±5.71	-			7.02±0.84	5.23±0.7*		24/45 (57.6%)		-	-
2	Agrawal et al. (24)	Metformin+CC (N=60)	27.38±3.92	25.45±3.22*	NS	HOMA-IR	2.83±1.29	1.62±0.59*	Y	20/60 (33.3%)	Y	16.60 (26.67%)	Y
		Metformin+MYO+CC (N=60)	27.71±3.60	25.77±3.48*			2.78±1.6	1.46±0.51*		38/60 (63.3%)		33/60 (55%)	
3	Ortega-González et al. (30)	Metformin (N=18)	34.1±1.6	32.9±1.7	Y	HOMA-IR	7.21±0.52	2.43±0.3	NS	3/18 (16.6%)	NS	2/18 (11.1%)	NS
		Pioglitazone (N=17)	32.2±1.0	34.0±1.2*			7.03±0.28	2.42±0.31		5/17 (29.4%)		2/17 (11.8%)	
4	Liu et al. (29)	24w Metf (N=80)	28.29±1.86	27.2±1.8*	Y	HOMA-IR	3.89±1.12	3.30±1.00	Y	15/80 (18.3%)	Y	-	-
		12w Exenatide switch to 12w Metf (N=78)	29.16±3.11	26.04±3.52*			4.21±1.89	2.92±1.31		34/78 (43.6%)		-	-
5	El Sharkwy and Sharaf El-Din (33)	Metf+CC (N=136)	34.4±3.4	32.5±3.2*	NS	HOMA-IR	2.83±0.7	2.22±0.34	Y	9/136 (6.6%)	Y	-	-
		Metf+CC+L-carnitine (N=138)	35.5±3.2	32.2±4.1*			2.72±0.38	1.8±0.43*		39/138 (28.2%)		-	-
6	Legro et al. (27)	Metformin (N=208)	35.6±8.5	-0.6±2.2	Y (Metf vs CC)	Change in HOMA-IR	5.6±8.9	+ (0.7±10.9)	Y (CC vs Metf+CC)	18/208 (8.7%)^a^	Y (Metf vs CC)	15/208 (7.2%)^a^	Y (Metf vs CC)
		CC (N=209)	36.0±8.9	+0.2±1.6	Y (Metf+CC vs CC)		5.2±5.3	+ (2.2±12.6)	NS (Other comparison	50/209 (23.9%)^b^	Y (Metf vs Metf+CC)	47/209 (22.5%)^b^	Y (Metf vs Metf+CC)
		Metformin+CC (N=209)	34.2±8.4	-0.5±1.4	NS (Metf+CC vs Metf)		5.6±10.2	- (0.1±11.8)		65/209 (31.1%)^b^	NS (CC vs Metf+CC)	56/209 (26.8)^b^	NS (CC vs Metf+CC)
7	Karimzadeh et al. (26)	Metformin (N=100)	28.80±3.18	28.45±2.8	NS	FPG/Insulin	4.67±0.9	6.07±1.4	Y	40/100 (40.0%)	Y	-	-
		Placebo (N=100)	29.49±4.75	29.29±4.8			5.03±1.3	5.05±1.3		11/100 (11.0%)		-	-
8	Yarali et al. (32)	Metformin (N=10)	28.6±4.0	28.0±3.4	NS	FPG/Insulin	0.10±0.03	0.12±0.07	NS	3/10 (30.0%)	NS	-	-
		Placebo (N=15)	29.6±4.8	29.8±4.9			0.09±0.04	0.10±0.06		1/15 (6.7%)		-	-
9	George et al. (25)	Metf+CC (N=30)	25.7±3.9	24.9±2.9*	Y	FPG/insulin	11.7±12.8	14.6±19.0	NS	5/30 (16.7%)	NS	-	-
		hMG (N=30)	25.9±2.9	25.9±2.9			4.8±2.7	11.6±6.5*		7/30 (23.3%)		-	-
10	Legro et al. (28)	Lifestyle modification (N=50)	35.1±4.6	-		Insulin sensitivity index	1.7 (1.0, 2.7)	+1.25 (1.09 –1.43)*	Y (Lifestyle modification vs OCPs	13/50 (26.0%)	NS	13/50 (26.0%)	NS
		OCPs (N=49)	35.1±4.2	-			2.0 (1.4, 2.6)	+0.89 (0.77–1.03)		7/49 (14.3%)		5/49 (10.2%)	(When Merge Life and combination group, Y)
		Combination (N=50)	35.5±4.4	-			1.9 (1.2, 3.2)	+1.21 (1.06 –1.39)*	Y (Combination vs OCPs)	13/50 (26.0%)		12/50 (24.0%)	

1, Mean±SD or Mean change (95% CI); 2, The significant change reported in the RCTs.

*, The change after treatment was significant compared with that in baseline.

Y, The difference between groups was significant.

NS, Non-significant.

+, Value increased compared with baseline.

-, Value decreased compared with baseline.

a, b, the group with label a was significantly different from the group labeled b, and the same label means non-significant change between groups.

BMI, Body mass index; CC, clomiphene citreate; hMG, human menopausal gonadotropin; Metf, Metformin; MYO, myoinositol; OCPs, Oral contraceptive pills; HOMA-IR, homeostasis model assessment of insulin resistance; FPG, fasting plasma glucose; IS, Insulin sensitivity.

Subjects could have been pregnant at any time during treatment, and therefore treatment was stopped when human chorionic gonadotropin level increased. Thus, treatment duration was defined as the longest treatment in a given trial. Treatment duration in the included trials was at least 3 months except for that in the report by Yarali et al. (32) (Trial No.8 in Tables) which was only up to 6 weeks. Among the 10 included RCTs, the longest treatment duration was up to around 6 months in five trials (Trial No.1, 3, 4, 6, 9 in Tables) (25, 27, 29–31). There were only two trials with relevantly large sample sizes (>100 subjects) and sufficiently long treatment duration (6 months or 24 weeks) (Trial No.4, 6) (27, 29) (**Table 1**).

**Figure 2:**
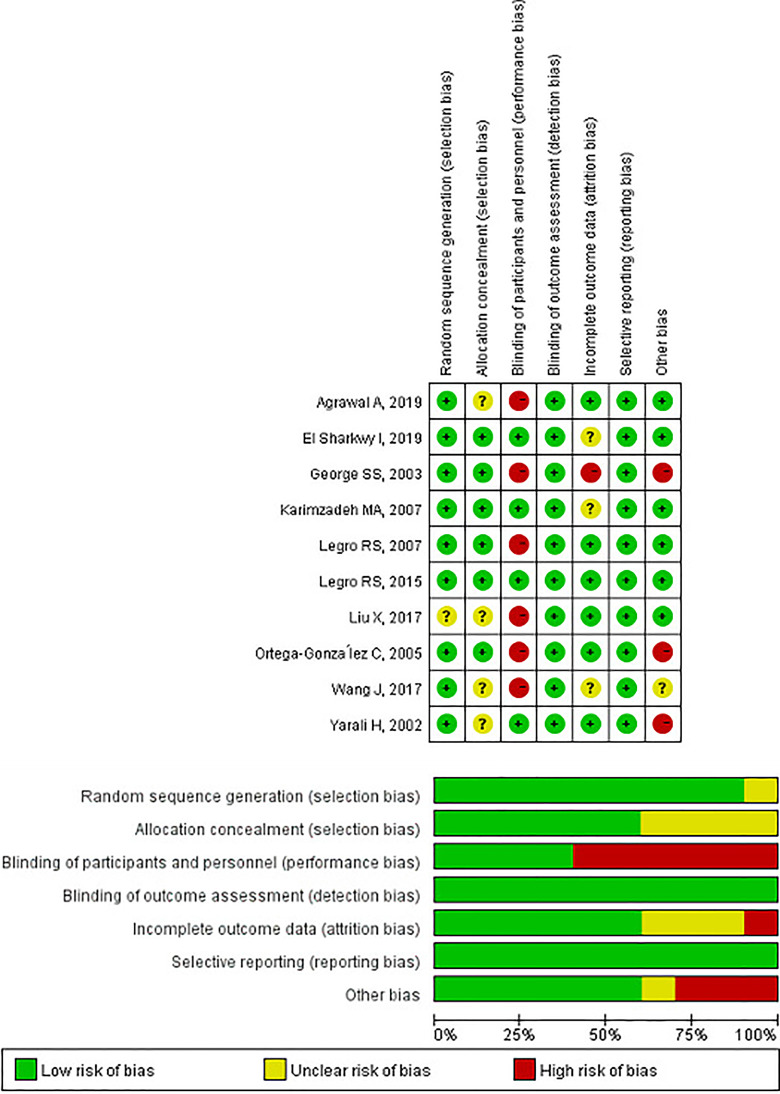
The cochrane collaboration risk of bias evaluation for the included studies.

Among the 10 included RCTs, one study did not describe the random sequence generation method (29), four trials did not describe the allocation concealment method (24, 29, 31, 32). Only three RCTs had a low risk of blinding participants and personnel (26, 32, 33), and Legro RS et al. could not use the blinding method due to lifestyle modification therapy (28). And because the outcome, clinical pregnancy rate, was not affected even without blinding method, all the ten studies had a low risk of outcome assessment. One study was rated high risk because of the high drop-out rate in the hCG group during follow-up (25) and three RCTs did not mention drop-out (26, 31, 33). All ten studies reported appropriate outcomes. Three studies were considered to have a high risk of other bias due to the small sample size (25, 30, 32), and one study was rated unclear risk because the sample size of the two groups was moderately different which were 33 in the metformin+CC group and 45 in the exenatide+CC group (31) (**Figure 2**).

## Discussion

### Improvement of IS After Treatment Increased Pregnancy Rate in Infertile PCOS Women Regardless of Nonsurgical Intervention

Obesity is highly involved in the development of IR (34). To ensure consistency in our analyses, the included trials were analyzed by classifying body weight according to BMI at baseline.

Among RCTs that reported HOMA-IR, there were two in which mean BMI of groups was classified as overweight (Trial No.1, 2 in Tables) (24, 31), two where subjects were obese (Trial No.3, 4 in Tables) (29, 30), and two where subjects were severely obese (Trial No. 5, 6 in Tables) (27, 33). In all these trials, baseline HOMA-IR was not significantly different between groups in the same trial. In trials where subjects were overweight or obese, pregnancy rate increased in the group that had a significantly larger reduction of HOMA-IR after treatment (24, 29, 31). Furthermore, if there were no significant differences in HOMA-IR between groups, there were no significant differences in pregnancy rate between groups (30). Agrawal et al. (24) (Trial No.2 in Tables) and Ortega-González et al. (30) (Trial No.3 in Tables) also reported live birth rate and arrived at the same conclusion as that for pregnancy rate. Regarding two other trials where subjects were severely obese, the same observation was made by El Sharkwy and Sharaf El-Din (33) (Trial No.5 in Tables), but not by Legro et al. (27) (Trial No.6 in Tables). In the trial by *El* El Sharkwy and Sharaf El-Din (33) (Trial No.5 in Tables), the group with the addition of L-carnitine to the combination of metformin and CC achieved a further reduction of HOMA-IR and higher clinical pregnancy rate (33). In the trial by Legro et al. (27) (Trial No.6 in Tables), the three groups were administered metformin monotherapy, CC monotherapy, or combined metformin plus CC. Treatments were provided for up to 6 months, and although none achieved reduction of HOMA-IR and even significantly increased in CC group, HOMA-IR in the combined therapy group was significantly lower than in the CC group. However, the significant difference in HOMA-IR was because of the increase in CC monotherapy group. Regarding pregnancy rate, there was no significant difference between the CC and CC plus metformin groups. However, the pregnancy rate in the metformin group was significantly lower than in the other two groups (26). In the trial by Legro et al. (27) (Trial No.6 in Tables), no treatments (CC alone, metformin alone, or combined CC plus metformin) achieved reduction of IR, which suggested pregnancy rate was more closely associated with the ovulation-promoting effect of CC. The explanation for why metformin failed to achieve improvement of IS in the trial may have been the severe obesity of subjects. However, more evidence is necessary to confirm this theory.

Therefore, these trials suggested pregnancy rate was increased in the groups with a larger reduction of HOMA-IR.

For parameters that reflect IS (FPG/INS ratio or insulin sensitivity index), three trials recruited overweight PCOS women (Trial No.7, 8, 9 in Tables) (25, 26, 32), whereas one had severely obese subjects (Trial No.10 in Tables) (28). In overweight subjects, the pregnancy rate was significantly higher in the group where FPG/INS ratio was significantly higher compared with other groups in the same trial (26). Moreover, when IS did not differ significantly between groups, there was no difference in pregnancy rates (25, 32). In the trial by Legro et al. (Trial No.10 in Tables), subjects were severely obese and received lifestyle modification, oral contraceptive pills (OCPs), or combined therapy with lifestyle modification and OCPs for 16 weeks (28). After treatment, insulin sensitivity index in the lifestyle modification and combined therapy groups was significantly increased and significantly higher than in the OCPs group. However, although pregnancy rate was not significantly different between the three groups, there was a trend toward a higher pregnancy rate in the lifestyle modification and combined therapy groups compared with the OCPs group. The authors believed this may have been because of the sample size. When they merged data from the lifestyle modification and combined therapy groups, they found the difference was significant between the merged and OCPs groups. This conclusion was also consistent with the previous discussion that pregnancy rate was increased in the groups with a larger improvement of IS. Live birth rate was reported by Legro et al. (28) (Trial No.10 in Tables), and the trend was the same as with pregnancy rate.

These results suggested pregnancy rate increased in the group with significant improvement of IS, even when the various parameters that reflect IS are considered.

All of these RCTs suggested achieving improvement of IS (including reduction of IR and increase of IS) after treatment is associated with increased pregnancy rate in infertile PCOS women, regardless of nonsurgical intervention. Furthermore, the benefit of post-treatment improvement of IS was more pronounced in women without severe obesity. For severely obese individuals, effective improvement of IS was difficult to achieve, which may have been because of inflammation and other issues caused by severe obesity.

### The Benefits of Improvement of IS was Inconsistent With the Reduction of BMI

Obesity is one of the most important risk factors for IR. However, it appears that improvement of IS after treatment was not consistent with the change of BMI in infertile PCOS women. BMI did not change after treatment compared with that before treatment and was not significantly different between groups in the trials by Agrawal et al. (24) (Trial No.2 in Tables), El Sharkwy and Sharaf El-Din (33) (Trial No.5 in Tables), and Karimzadeh et al. (26) (Trial No.7 in Tables). However, HOMA-IR or FPG/INS ratio was significantly changed, and pregnancy rate was different between groups in the three RCTs (24, 26, 33).

These data indicated that the reproductive benefit from the improvement of IS might be more important than that in the reduction of BMI.

### PCOS Women Without Significant Insulin Resistance at Baseline Also Benefit From the Improvement of IS

To compare IS status in the different trials, significant IR was defined in this review as group mean HOMA-IR score >3.0, based on a large RCT where insulin resistance interventions after stroke were explored, and IR was identified as HOMA-IR score >3.0 (35). Although there are no globally standardized stratification criteria for the degree of IR, comparisons and analyses can aid in identifying evidence and trends regarding the effects of improvement of IS. HOMA-IR was reported in six studies, two of which included patients without IR (i.e., group mean HOMA-IR score <3.0) (Trial No.2, 5 in Tables). In the two studies, intervention groups achieved an improvement of IS, and the group with a larger reduction of HOMA-IR demonstrated a higher pregnancy rate.

This suggested improvement of IS can also increase pregnancy rate in PCOS women without IR. However, large, well-designed RCTs should be conducted to further validate this observation.

### Benefits of Improvement of IS on Reproductive Outcomes Occurred With Various Nonsurgical Interventions but Were Not Dependent on Insulin Sensitizers

Two of the included RCTs evaluated the effect of metformin compared with placebo (26, 32). Treatment with metformin for 3 months in the trial by Karimzadeh et al. (26) (Trial No.7 in Tables) improved IS and increased pregnancy rate (26). This was consistent with the conclusion from a meta-analysis (13). However, 6-week metformin therapy in the trial by Yarali et al. (Trial No.8 in Tables) did not improve IS, and no difference was found between the metformin and placebo groups (32). This may have been because 6 weeks was too short to achieve significant improvement of IS.

Regarding combined therapy with metformin and CC, the meta-analysis did not find it was superior to CC monotherapy in PCOS infertile women, either in those with BMI<30.0 or BMI>30.0 (13). Similar observations were noted as part of this review. According to Legro et al. (27) (Trial No.6 in Tables), the combined therapy group demonstrated trends toward lower HOMA-IR and higher pregnancy rate compared with the CC monotherapy group, although they were not significant (28). This suggests the benefits of improvement of IS may be the secondary result of the potent effects of CC and other ovulation-inducing agents.

Other insulin sensitizers were also shown to effectively improve IS. Exenatide, a glucagon-like peptide-1 receptor agonist, was reported to reduce IR in type 2 diabetes patients (36). Compared with metformin, exenatide improved IS and pregnancy rate to a higher degree (29, 31). Wang et al. (31) (Trial No.1 in Tables) and Liu X et al. (29) (Trial No.4 in Tables) showed that exenatide improved IS and pregnancy rate, and the effect was superior to that of metformin, despite exenatide and metformin both being insulin-sensitizing agents. Pioglitazone, a typical insulin sensitizer, was proven superior to metformin at reducing IR (37, 38). However, other trials reported no difference between pioglitazone and metformin in improving IS in individuals with nonalcoholic fatty liver disease (39) or PCOS (40). Among the ten included RCTs, only one compared pioglitazone with metformin in PCOS infertile women, although no significant difference was found between the two groups (Trial No.3 in Tables) (30). However, this may have been because the sample size (N=47) was too small to demonstrate a statistical difference. More evidence is necessary to confirm the efficiency and safety of pioglitazone in PCOS women.

Adjuvant drugs, such as myoinositol (MYO) and L-carnitine, were not considered as traditional insulin-sensitizing agents but helped improve IS in PCOS women (Trial No.2, 5 in Tables) (24, 33). MYO is the precursor of inositol triphosphate, a second messenger that regulates thyroid-stimulating hormone, follicle-stimulating hormone, and insulin (41). A previous study indicated that MYO has an effect on improving IS (42) and estradiol levels in PCOS women (43). Previous studies suggested L-carnitine can reduce IR and BMI in PCOS women (44, 45). However, additional high-quality RCTs are required to confirm the effect and safety of these drugs in infertile PCOS women.

Only one RCT (with a small sample size (N=149) and short-term intervention) assessed the effect of lifestyle modification in infertile women with PCOS (Trial No.10 in Tables) (28). However, it was found that short-term lifestyle modification improved IS and increased pregnancy and live birth rates (28). Lifestyle modification may be among the most cost-efficient methods for significantly improving IS and reducing BMI, although clinical compliance is poor. All overweight or obese women should undergo lifestyle modification as a basic intervention.

To summarize, metformin and other related drugs, or lifestyle modification can increase pregnancy rate, but only if these interventions significantly improve IS. It appears exenatide had a better effect than metformin at increasing pregnancy rate and improving IS. Metformin has been proven safe and is nonteratogenic, but additional evidence is necessary to confirm the safety of other insulin sensitizers (46).

### Strengths and Limitation

By screening and analyzing existing RCTs, improvement of IR following nonsurgical interventions was strongly correlated with increased pregnancy rate in infertile women with PCOS. Although only two RCTs, Liu et al. (29) and Legro et al. (27) (Trial No.4,6), had relevantly large sample sizes and sufficient treatment durations, the conclusion and analysis were consistent in the studies. They were able to determine that pregnancy rate increased in the groups that achieved an improvement of IS. Therefore, nonsurgical therapeutic strategies that result in superior improvement of IS may aid infertile PCOS women to increase their possibility of a successful pregnancy.

This review had limitations. Evidence demonstrated that IUGR, SGA, and history of family diabetes contributed to PCOS patients with IR, and these factors might also affect the effectiveness of treatment on improving insulin sensitivity. However, few trials concerned about the factors so that there was insufficient information that can be obtained and discussed in the review. More evidence is required to fill the gap. Besides, most RCTs that explored the reproductive effects of nonsurgical interventions in PCOS women did not report changes in IR. Therefore, only some of these studies were included in this review, and some had small sample sizes. High-quality and large sample size RCTs are needed to confirm the results discussed in the systemic review.

## Conclusion

Among the various nonsurgical interventions, the benefit of improvement of IS appeared to be superior in PCOS women without severe obesity. The benefit of improvement of IS appears to be more important than that in changes of BMI after treatment even the BMI is one of the most crucial factors for IS. And IS improvement also benefits PCOS women without IR at baseline. Metformin and other related drugs, and lifestyle modification, may also be capable of improving IS. It appears exenatide had a better effect than metformin at improving IS and increasing pregnancy rate. Although additional large, well-designed RCTs are necessary to confirm the benefits, the review emphasized the importance of achieving IS improvement in infertile PCOS women treatment.

## Data Availability Statement

The original contributions presented in the study are included in the article/supplementary material. Further inquiries can be directed to the corresponding author.

## Author Contributions

YL provided the conception, searched the literature, participated in planning how to do the work, abstracting study details and writing the manuscript. JL participated in screening and abstracting study details, discussing and organized the main text and writing the manuscript. ZY provided the conception, set the inclusion/exclusion criteria, participated in discussing and organizing the main text and revised the manuscript. DL provided clinical advice during the whole work in the view of gynecology and obstetrics. JM screened the studies. NT planed the whole work and revised the manuscript. All authors contributed to the article and approved the submitted version.

## Conflict of Interest

The authors declare that the research was conducted in the absence of any commercial or financial relationships that could be construed as a potential conflict of interest.
